# Development and psychometric properties of the Ageism Questionnaire for Nurses (AQN)

**DOI:** 10.1186/s12912-026-05198-3

**Published:** 2026-08-06

**Authors:** Mingtu Xu, Bo Wu, Cong Fu, Hongyang Tian, Tiancheng Mo, Guangying Zhu, Liu Liu, Runhui Tian

**Affiliations:** 1 Department of Mental Health, The First Hospital of Jilin University, Changchun, Jilin China; 2 Department of Mental Health, The Sixth Hospital of Changchun, Changchun, Jilin China

**Keywords:** Ageism, Nurses, Social exclusion, Factor analysis

## Abstract

**Background:**

With the accelerating aging of Chinese society, nursing professionals are progressively tasked with providing care for a growing population of elderly patients annually. Concurrently, manifestations of ageism within this professional cohort have become increasingly salient, underscoring an urgent imperative for empirical investigation into this phenomenon within healthcare contexts.

**Objective:**

This study aimed to develop and validate a theoretically grounded Ageism Questionnaire for Chinese Nurses (AQN), tailored to actual clinical nursing scenarios.

**Method:**

Initial questionnaire items were formulated through a synthesis of extant literature and semi-structured interviews with nursing practitioners. Subsequent refinement and item screening were conducted via the Delphi method. Data were collected between January and March 2025 from nurses engaged in elderly patient care across six public hospitals in Jilin Province.Data were collected using a sociodemographic sheet, Ageism Questionnaire for Nurses, the Fraboni Scale of Ageism, and the Utrecht Work Engagement Scale.The psychometric properties of the AQN, encompassing structural validity, content validity, criterion validity, test-retest reliability, and internal consistency reliability, were rigorously evaluated.

**Results:**

This study finally developed the Ageism Questionnaire for Chinese Nurses (AQN) with 2 dimensions and 23 items. Exploratory factor analysis verified the dimensions of ignoring elderly patients and rejecting elderly patients in the questionnaire. Confirmatory factor analysis showed that the two-dimensional structure of the questionnaire had a good fit. The alpha coefficients for the ignoring elderly patients dimension, rejecting elderly patients dimension, and total score were 0.907, 0.973, and 0.967, respectively. The split-half reliability was 0.864, 0.953, and 0.893, and the retest reliability was 0.889, 0.838, and 0.883, respectively. The criterion validity test results showed that the ignoring elderly patients dimension, rejecting elderly patients dimension, and total ageism attitude score were significantly correlated with work engagement.

**Conclusion:**

The AQN exhibits robust psychometric properties, positioning it as a reliable and valid instrument for assessing ageist attitudes among nurses in Chinese healthcare settings.

**Supplementary Information:**

The online version contains supplementary material available at https://doi.org/10.1186/s12912-026-05198-3.

## Introduction

Population aging has emerged as a persistent global phenomenon, with numerous Asian nations transitioning into distinct phases of societal aging [1–2]. According to the National Bureau of Statistics of China, individuals ≥ 65 accounted for 15.4% of the population, respectively, by the end of 2023, signifying entry into a “aged society” [3]. This demographic shift, compounded by rising life expectancy, has precipitated an exponential demand for healthcare services. The 2023 China Health Statistics Yearbook reflects the substantial demand for hospital services among old adults: 45.8% of male and 38.7% of female hospital discharges in 2022 involved patients aged ≥ 60 [4]. Elderly patients often present with multiple chronic conditions, which significantly increases the workload of nursing staff in daily care and further exacerbates systemic pressure on the entire healthcare system [5, 6].

Nurses constitute the cornerstone of healthcare systems, representing over half of the global healthcare workforce [7]. Prolonged frontline interactions with elderly patients, however, render nurses uniquely susceptible to occupational stressors. Notably, elderly inpatients frequently present with complex comorbidities, which may exacerbate nurses’ susceptibility to burnout and negative affective responses, including resistance or aversion [8]. Existing studies suggest that ageism can, to some extent, explain these negative emotions and behaviors among nurses toward the elderly patients [9]. In the fields of sociology and psychology, ageism refers to the unconscious perception of old adults as physiologically or socially weaker within a social group, leading to discriminatory attitudes and behaviors toward them. Similar to other forms of discrimination (e.g., racism or discrimination against sexual minorities), ageism specifically denotes prejudice and stereotyping against old adults, often involving assumptions about certain characteristics (e.g., rigid thinking or lack of creativity) based on prior experience rather than factual evidence [10–11]. Stereotypes reflect neutral cognitive attitudes, prejudice embodies negative affective, while discrimination primarily manifests in individual behaviors. Notably, both stereotypes and prejudice toward old adults may constitute critical components in the formation of ageism [12].

Numerous studies have highlighted the widespread presence of ageism nursing education and clinical practice(this domain). Surveys on career preferences among nursing students reveal prevalent ageist attitudes, with many students unwilling to work in geriatric care due to perceptions of old adults as inflexible, difficult to communicate with, unable to live independently, or having poor hygiene [13]. Only 5.1% of nursing students expressed a willingness to pursue geriatric nursing as a future career [13–14]. Studies involving old adults have also found that 29% of old adults reported experiencing discrimination from healthcare providers during medical visits, with 5.9% feeling discriminated against at every visit [15]. Qualitative research on ageism has indicated that nurses exhibit significant ageist attitudes toward elderly patients who are bedridden or have cognitive impairments, often believing that these patients do not require the same level of assessment and treatment as younger patients. Additionally, elderly patients in frontline nursing care often present with high demands and complex care needs [16]. Quantitative studies show that 54% of healthcare workers exhibit ageist attitudes in their experiences caring for old adults, with nurses’ ageism manifesting not only in neglecting elderly patients’ needs but also in abusive behaviors toward them [17].

Research has noted that ageism among nurses is highly evident in the nursing process. For instance, nurses’ negative attitudes result in short, superficial conversations with elderly patients, and they are often unwilling to discuss medical conditions in depth with them [18]. Qualitative studies have further identified that ageism among nurses manifests as inappropriate care behaviors, such as perceiving care as overly difficult or communication as challenging [19]. More critically, the emergence of ageism affects old adults health. A study involving old adults across 45 countries found that ageism impacts 11 health domains, including impaired functioning, chronic illnesses, and mental illness [20]. In healthcare settings, ageism not only affects the treatment of physical conditions in elderly patients but also contributes to mental health issues such as depression and anxiety [21]. Given the detrimental effects of ageism and its particular relevance in nursing, the phenomenon of ageism among nurses in their work warrants greater attention.

Regardless of whether in the field of nursing education or nursing practice, considerable quantitative research has been conducted on ageism [14–23]. In previous studies, the Kogan’s Attitudes Toward Old People Scale (KAOP) and the Relating Older People Evaluation (ROPE) have been used as measurement tools for ageism [24–25]. The KAOP is a mature and widely utilized instrument designed to assess individuals’ prejudice and stereotypes toward old adultsm, Furthermore, the original target population of this instrument was general university students, and its item content, including both the measurement approaches and the evaluative statements, is grounded in everyday social interactions.For example, “Once elderly people have the means, they are willing to give up work” and “Elderly people always disapprove of young people” are not applicable to nursing care contexts [24]. The ROPE scale, on the other hand, was developed based on scenarios derived from nursing students’ daily interactions with the old adults.Consequently, some items are also not applicable to nursing care contexts, for example, “sending birthday cards that joke about elderly people’s age” and “holding the door open for older individuals.” [25].

The classic Fraboni Scale of Ageism (FSA), a frequently utilized quantitative tool for assessing ageism, was developed by Fraboni, Saltstone, and Hughes in 1990. It consists of three dimensions (antilocution, avoidance, and discrimination) with a total of 29 items, rated on a 4-point scale [26]. This scale has been extensively studied in the nursing field and validated across various cultural contexts [27–30]. Existing scales primarily assess stereotypes and prejudice toward old adults among the general population, focusing predominantly on attitudes and perceptions toward the old adults. While some scales have been validated with nursing staff, certain items lack relevance to healthcare contexts. For instance, the Fraboni Scale of Ageism (FSA) includes items such as ‘Special athletic events should be arranged for old adults so they can compete at their own level’ - these items emphasize general social cognition and affect rather than specific nursing scenarios. Consequently, such scales demonstrate limited clinical utility in practical applications like nursing staff training interventions or hospital management evaluations [29–31].

The Chinese government’s Guidelines on Deepening the Reform and Development of Elderly Care Services explicitly addresses workforce development and social participation in elderly care. Confronting the global challenge of ageism, effectively addressing age discrimination carries profound implications for old adults’ health and well-being. Grounded in relevant theoretical frameworks and incorporating clinical nursing staff’s practical clinical experiences with older patients, this study developed a specialized ageism questionnaire tailored for healthcare providers [32].

## Methods

This study was theoretically anchored in psychometric principles and methodologically designed to develop a validated instrument for assessing nurses’ rejection behaviors toward elderly patients in clinical healthcare contexts. The research was structured into three sequential phases.Phase 1:established the preliminary questionnaire framework and item pool by integrating theoretical foundations, expert panel evaluations, and frontline nurses’ practical experience; Phase 2:employed the Delphi method for iterative expert consultations to refine and screen scale items; Phase 3:we conducted a cross-sectional survey for data collection, followed by exploratory factor analysis to examine the dimensional structure of the refined scale, and comprehensive psychometric evaluations including reliability testing and validity assessments to establish the scale’s internal consistency, construct validity, and criterion-related validity.

For the purpose of psychometric validation, data collection was conducted from November 2024 to January 2025 as Phase 3 of this study across three tertiary hospitals in Jilin Province, with the following participant criteria: inclusion criteria were currently employed nursing staff directly involved in elderly care with ≥ 12 months of clinical experience who voluntarily participated and provided signed informed consent; exclusion criteria included non-direct care staff, those with less than 12 months of probationary experience, individuals who had recently experienced major life events, those with severe mental disorders, and participants with incomplete questionnaire responses. The study was conducted through the Wenjuanxing online survey platform using the following procedures: (1) targeted distribution of questionnaire links to nursing staff groups in partner medical institutions, supplemented by recruitment through professional nursing WeChat public accounts; (2) automatic exclusion of ineligible participants through screening questions (e.g., work experience, position type) embedded in the questionnaire; (3) elimination of abnormal responses such as excessively short completion times or identical answers to all questions.Ethical oversight was ensured through formal approval by the Institutional Review Board of the First Hospital of Jilin University (AF-IRB-032-06), with strict adherence to standardized research governance protocols.

### Theoretical framework

The Social Exclusion Theory, initially proposed in the 1960s, was primarily employed to examine social inequality issues. With the deepening of research, its conceptual framework has been continuously expanded and gradually applied to explain the marginalization phenomenon during the aging process [33–34]. The theory posits that when individuals are excluded by social groups, their needs for belonging and relationships encounter systematic barriers. In aging research, ageism is considered a significant contributing factor to social exclusion among old adults, while social exclusion behaviors also represent crucial external manifestations of ageism [35–36]. Within the specific context of nursing care, the long-term interactive relationships between healthcare providers and elderly patients make social exclusion phenomena particularly noteworthy. In nursing settings, social exclusion primarily manifests in two forms: neglect and rejection [36]. In nursing practice, ageism-related neglect manifests through delayed responses to older patients’ needs and insufficient attention to detail in care procedures, demonstrating both passive and implicit characteristics. [37–38]. Ageism-related rejection manifests through active avoidance or resistance behaviors during care delivery, exhibiting overt and intentional characteristics [39]. These exclusionary behaviors reflect the presence of ageism among nursing staff, which not only directly compromises the quality of care but may also exert profound negative impacts on older patients’ psychological well-being. [40–41]

Guided by the analytical framework of Social Exclusion Theory, this study operationalizes nurses’ discriminatory behaviors toward older patients into two specific dimensions: neglect and rejection of elderly patients. By developing specialized measurement tools, the research aims to systematically identify and evaluate ageism phenomena in nursing practice, thereby providing scientific evidence for improving care quality and developing targeted interventions. The selection of this theoretical framework not only demonstrates robust theoretical grounding but also precisely captures the unique attitudinal and behavioral manifestations of ageism within the distinctive context of nursing care.

## Instrument development

### Phase 1: Item formation

This study developed an ageism questionnaire with “neglect” and “rejection” dimensions based on Social Exclusion Theory through literature analysis, clinical observations, and expert consultation. The initial item pool was derived from existing ageism scales and expert recommendations based on literature and clinical geriatric experience. Our interdisciplinary team (4 psychiatry/clinical medicine professors, 4 nursing researchers, and 2 psychology researchers) created the preliminary Ageism Questionnaire for Nurses (AQN) by integrating theoretical frameworks with clinical practice, ultimately retaining 26 items after removing duplicates, merging similar items, and optimizing wording.

To further enrich the item pool and ensure its relevance to frontline practice, we conducted semi-structured interviews with 10 nurses who had at least 12 months of experience caring for elderly patients. Each interview lasted 15–30 min and followed a topic guide (see supplementary material 1 for the complete interview outline). Two researchers independently reviewed the transcripts to extract recurring behavioral examples, which were then mapped to the two theoretical dimensions—neglect and rejection. The team reformulated these examples into clear, first-person, action-oriented statements and retained items only upon consensus. Through this procedure, eight additional items were generated, including three items (item12, item13, item14) related to ignoring behaviors and five items (item30, item31, item32, item33, item34) related to rejection behaviors, resulting in a 34-item preliminary version for subsequent Delphi evaluation.

### Phase 2: Delphi evaluation

This study employed a standardized Delphi expert consultation method for questionnaire item screening and revision. Regarding expert selection, we invited 12 specialists with extensive experience in geriatric care, comprising: (1) 4 nursing experts holding doctoral degrees with over 5 years of geriatric care research experience, (2) 4 nursing administrators with more than 10 years of clinical management practice, and (3) 4 psychology professors specializing in elderly mental health research for at least 3 years.In the end, ten experts accepted our invitation. (For detailed information on all experts, please refer to supplementary material 2). Using a 5-point Likert scale (1 = not important, 5 = extremely important), the experts rated item importance and provided revision suggestions. Items with mean importance scores below 4 or coefficient of variation (CV) exceeding 0.25 were eliminated [42].

The Delphi process was conducted online via email. Experts had two weeks per round to respond, with a reminder after the first week. All responses were anonymized. A three-week interval between rounds allowed for data analysis and item revision. In the second round, experts received the revised questionnaire along with a summary of first-round results (mean scores and CV values) to guide their reassessment.In the first-round expert consultation, all 10 invited experts completed the evaluation with an authority coefficient (Cr) ranging from 0.9 to 1.0, which was calculated using the formula Cr = (Ca + Cs) / 2 and indicated high professional credibility.Using a 5-point rating scale, experts assessed questionnaire items based on predefined criteria, resulting in the elimination of 8 items with CV values exceeding 0.25 and mean importance scores below 4. Experts identified limitations in specific items (e.g., Item 2 “Having poor attitudes toward elderly patients” was considered overly vague; Item 8 “I neglect elderly patients’ emotional needs” was deemed too general and clinically misaligned). They also proposed wording improvements (e.g., Item 27,”In my nursing practice, I actively avoid interactions withelderly patients” was modified to the more behaviorally specific “In my nursing work, I intentionally avoid interacting with elderly patients to reduce contact with them.”following recommendations that items should clearly reflect concrete behaviors or attitudes). The Kendall’s W coefficient was 0.710 (*P* < 0.01), showing strong consistency. In the second round, all 10 experts reevaluated the revised items, with retained items scoring 4.6-5.0 (CV: 0-0.11) and Kendall’s W of 0.153 (*P* < 0.05), demonstrating consensus. The final 26-item questionnaire achieved optimal content validity and clinical applicability standards.

### Phase 3: Psychometric validation

In the exploratory factor analysis (EFA) phase (Sample A), we recruited eligible participants from two public hospitals in Jilin Province, China, using convenience sampling. All eligible participants signed informed consent forms. In the confirmatory factor analysis (CFA) phase (Sample B), we further validated the items that passed the EFA. For this phase, we used convenience sampling again to recruit participants from four additional public hospitals in Jilin Province. Previous studies suggest that each item should be assigned 5–10 participants. In this study, 402 participants were included in the EFA, and 760 participants were included in the CFA.

The items that passed both EFA and CFA were subjected to reliability and validity testing. Validity was assessed using criterion-related validity, with the sample drawn from Sample B. Based on prior studies, we employed the Ageism Questionnaire and the Work Engagement Scale as criterion measures. Reliability was evaluated using the Cronbach’s α coefficient, split-half reliability, and test-retest reliability. The α coefficient and split-half reliability were calculated using data from Sample B. Additionally, test-retest reliability was assessed by re-administering the questionnaire to a subset of Sample B participants after a two-week interval.A total of 102 valid questionnaires were collected for the test-retest reliability assessment in this study.

### Ethics statement

This study was approved by the Ethics Committee of the First Hospital of Jilin University (AF-IRB-032-06). We followed the ethical principles established in the Declaration of Helsinki, which ensure respect for each individual and his/her self-determination. Prior to the survey, the participants were given sufficient information about the study’s purpose, the data’s intended use, and the data’s protection. All participants provided informed consent to participate in the study.

## Measures

### Self-developed demographic questionnaire

A survey was conducted to collect basic demographic information from nurses, including gender (1-male, 2-female), marital status (1-unmarried, 2-married, 3-divorced, 4-widowed), education level (1-high school or vocational school, 2-junior college, 3-university, 4- graduate or above), professional title (1-nurse, 2- junior nurse, 3 -senior nurse, 4- deputy chief nurse or above), years of work experience (1-less than 5 years, 2–6–10 years, 3–11–15 years, 4–16–20 years, 5-more than 20 years), and frequency of contact with elderly patients (1-never, 2-occasionally, 3-frequently, 4-always).

### Ageism questionnaire

This study utilized the Chinese version of the Fraboni Scale of Ageism (FSA) to assess nurses’ attitudes toward ageism. The questionnaire comprises three dimensions: Exclusion, Stereotype, and Avoidance. It has been widely applied among nursing staff. In this study, the Cronbach’s α coefficient was 0.872 [29, 30].

### Work engagement scale

The Utrecht Work Engagement Scale (UWES-9) is a commonly used tool to measure work engagement, designed to evaluate employees’ enthusiasm, dedication, and vigor in their work. This scale has been extensively employed in studies of work engagement among nurses. In this study, the Cronbach’s α coefficient was 0.953 [43, 44].

### Statistical analysis

In this study, the Kaiser-Meyer-Olkin (KMO) test and Bartlett’s Test of Sphericity (χ²) were used as indicators for exploratory factor analysis (EFA). A KMO value greater than 0.7 and a Bartlett’s Test of Sphericity χ² with a p-value less than 0.05 indicate that the questionnaire items are suitable for EFA. For confirmatory factor analysis (CFA), the following indices were used: chi-square/degree of freedom ratio (χ²/df), comparative fit index (CFI), Tucker-Lewis index (TLI), standardized root mean square residual (SRMR), and root mean square error of approximation (RMSEA). The criteria for acceptable fit were: χ²/df < 5, CFI > 0.9, TLI > 0.9, SRMR close to zero, and RMSEA < 0.08.

For reliability testing, we examined both internal consistency reliability and test-retest reliability. Internal consistency reliability was assessed using Cronbach’s *α* coefficient, with a minimum acceptable threshold of 0.8. Test-retest reliability required a correlation coefficient of at least 0.7. For validity testing, we additionally evaluated criterion validity.

In this study, exploratory factor analysis (EFA), internal consistency reliability, split-half reliability, test-retest reliability, and criterion validity were analyzed using SPSS 24.0, while confirmatory factor analysis (CFA) was conducted using AMOS.

## Results

### Descriptive statistics of psychometric properties

Demographic data were collected from 1,200 questionnaires administered to nurses in Jilin Province, China. Among these, 1,162 responses met validity criteria, resulting in a response rate of 96.3%. The sample exhibited a mean age of 35.07 years (SD = 6.37). In the self-developed instrument assessing nursing practice engagement with elderly patients (scaled from 1 = “never” to 5 = “always”), participants demonstrated substantial interaction frequency, as evidenced by a mean score of 4.66 ± 0.57. This finding suggests a consistently high level of geriatric care involvement within the study cohort.As shown in Table 1.

**Table 1:** Participants characteristics

Measure（N=1162）	M ±SD/n（%）
Age（years）	35.07±6.37
Gender	
1. Male	102
2. Female	1060
Marital Status	
1. Single/Never married	242（20.8）
2. Married	901（77.5）
3. Divorced	18（1）
4. Widowed	1（0.1）
Educational Background	
1. High school	3(0.3)
2. Associate degree	86(7.4)
3. Bachelor's degree	1032(88.8)
4. Master's degree or higher	41(3.5)
Professional Title	
1. Staff nurse	126(10.8)
2. Nurse practitioner	297(25.6)
3. Nurse supervisor	689(59.3)
4. Associate chief nurse or above	50(4.3)
Years of Work Experience	
1. <5 years	192（16.5）
2. 6-10 years	231（19.9）
3. 11-15 years	516（44.4）
4. 16-20 years	115（9.9）
5. >20 years	108（9.3）
Extent of Contact with Elderly Patients	4.66±0.57

### Exploratory Factor Analysis (EFA)

The exploratory factor analysis (EFA) demonstrated robust suitability for dimensionality reduction, as indicated by a Kaiser-Meyer-Olkin (KMO) value of 0.971 and a statistically significant Bartlett’s Test of Sphericity (*p < 0.001*). Factors were extracted based on the eigenvalue-greater-than-1 criterion, employing an oblique rotation method (promax) to account for potential inter-factor correlations. Initial factor extraction revealed cross-loading items (Items 15, 17, and 18), which exhibited loadings exceeding the 0.4 threshold in multiple dimensions. These items were consequently eliminated to enhance factorial clarity.Post-removal, the KMO value remained high (0.969), and Bartlett’s Test of Sphericity retained statistical significance. The refined EFA yielded two interpretable factors with eigenvalues exceeding 1.0, collectively explaining 65.975% of the total variance. These factors were operationally defined as Neglect of Elderly Patients and Rejection of Elderly Patients, with detailed loadings and variance contributions tabulated in Tables 2 and 3, respectively.As shown in Table 2.

**Table 2:** Revised exploratory factor analysis(EFA)

	Neglect of Elderly Patients	Rejection of Elderly Patients
1	0.913	
3	0.695	
5	0.641	
6	0.792	
7	0.742	
9	0.600	
10	0.698	
11	0.602	
13	0.527	
14	0.554	
19		0.463
20		0.708
21		0.617
24		0.875
25		0.789
26		0.887
27		0.988
28		0.891
29		0.885
30		0.906
31		0.847
33		0.795
34		0.886

### Confirmatory factor analysis(CFA)

**Fig. 1:**
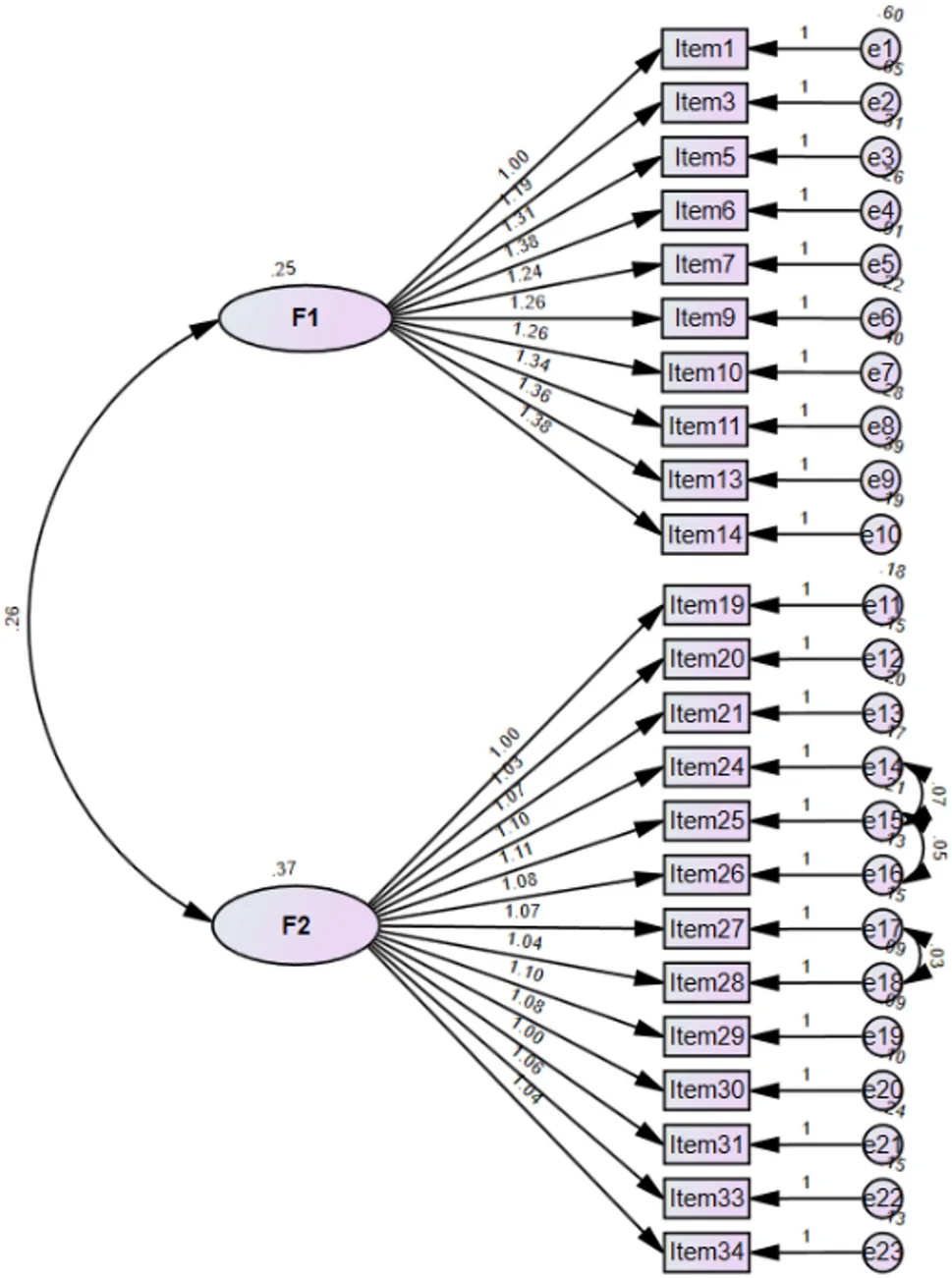
Structure equation modeling of Ageism Questionnaire for Nurses. Note: F1 = Neglect of Elderly Patients, F2 = Rejection of Elderly Patients. Item1-34 see Table 3

Confirmatory factor analysis (CFA) was conducted on 760 questionnaires in this study. After adjustments (e14-e15, e15-e16, e17-e18), the model demonstrated good fit indices. The χ²/df value was 4.639, and the RMSEA was 0.069. Additional fit indices included RMSEA = 0.069, TLI = 0.944, CFI = 0.950, IFI = 0.950, RFI = 0.930, NFI = 0.937, and RMR = 0.024. These indices meet the fit requirements, indicating a relatively good model fit.The structural equation model of the scale is presented in Fig. 1.

### Criterion validity

Criterion validity testing was conducted on Sample B. The results showed that the total score of ageism in nursing, the “Neglect of elderly patients” subscale, the “Rejection of elderly patients” subscale, and the total ageism attitude score were significantly positively correlated with each other. Meanwhile, the total score of ageism in nursing, the “Ignore of elderly patients” subscale, and the “Rejection of elderly patients” subscale were each negatively correlated with work engagement.As shown in Table 3.

### Reliability testing

Reliability testing was conducted on Sample B using Cronbach’s α coefficient. The results showed that the “Ignore of elderly patients” subscale (10 items) had an α coefficient of 0.907, the “Rejection of elderly patients” subscale (13 items) had an α coefficient of 0.973, and the overall scale had an α coefficient of 0.967. The split-half reliability was 0.864 for the “Ignore of elderly patients” subscale, 0.953 for the “Rejection of elderly patients” subscale, and 0.893 for the total scale.Test-retest reliability was assessed after a two-week interval, with results indicating a test-retest reliability of 0.889 for the “Ignore of elderly patients” subscale, 0.838 for the “Rejection of elderly patients” subscale, and 0.883 for the total scale.

**Table 3:** The indicators criterion-related validity

Ageism for Nurses	Ageism for Nurses	Neglect of Elderly Patients	Rejection of Elderly Patients	Ageism	Work Engagement
	-				
Neglect of Elderly Patients	0.934^**^	-			
Rejection of Elderly Patients	0.959^**^	0.794^**^	-		
Ageism	0.277^**^	0.250^**^	0.273^**^	-	
Work Engagement	-0.151^**^	-0.130^**^	-0.154^**^	-0.167^**^	-

^*^P < 0.01,^**^P < 0.001

### Ageism questionnaire in nursing

The Ageism Questionnaire in Nursing is presented in Table 4. Dimension 1 is “Ignore of elderly patients,” and Dimension 2 is “Rejection of elderly patients.” The scoring is as follows: 1 = never, 2 = occasionally, 3 = sometimes, 4 = frequently, 5 = always. The total score is the sum of all items, with a range from 23 to 115. Higher scores indicate a greater degree of ageism among nurses in their nursing work.

**Table 4:** Ageism questionnaire for nurses

Domain	Item text
Neglect of Elderly Patients	1. In my nursing work, I accidentally overlook the needs that elderly patients express.
	3. In my nursing work, I sometimes the help that elderly patients ask for.
	5. In my nursing work, I feel like the complaints from elderly patients don’t really matter.
	6. In my nursing work, I’m not careful enough when taking care of elderly patients.
	7. In my nursing work, I often don’t have the patience to listen to elderly patients fully describe their condition.
	9. In my nursing work, I sometimes neglect the safety of elderly patients.
	10. In my nursing work, I sometimes fail to pay attention to the dietary needs of elderly patients in time.
	11. In my nursing work, I rarely think about the mental state of elderly patients.
	13. In my nursing work, I don’t pay enough attention to some changes in the physical condition of elderly patients.
	14. In my nursing work, I don’t respond quickly enough to the reactions and feelings of elderly patients.
Rejection of Elderly Patients	19. During shift handovers in my nursing work, important care information about elderly patients gets missed or isn’t passed on accurately.
	20. In my nursing work, I sometimes feel that the health problems of elderly patients aren’t worth spending too much time or energy on.
	21. In my nursing work, when elderly patients make requests that go beyond my nursing duties, even if they are not too complicated, I still show resistance.
	24. In my nursing work, I find the demands of elderly patients annoying, so I sometimes avoid interacting with them.
	25. In my nursing work, the behavior of elderly patients can be hard for me to handle, leading me to reject their requests or avoid them.
	26. In my nursing work, I sometimes act cold or distant toward the emotional needs of elderly patients.
	27. In my nursing work, I intentionally avoid interacting with elderly patients to reduce contact with them.
	28. In my nursing work, I get irritated because of elderly patients’ chronic illnesses, which makes me show a rejecting attitude toward their care.
	29. In my nursing work, when elderly patients need help with something really troublesome, I might refuse to respond to their requests.
	30. In my nursing work, I show obvious reluctance or impatience.
	31. In my nursing work, I sometimes reject the non-medical needs of elderly patients (like needing company or help contacting family).
	33. In my nursing work, I feel unhappy whenever I find out that the patients I’m assigned to are older.
	34. In my nursing work, because communication can be such a hassle, I resist answering questions from elderly patients

## Discussion

This study developed the Ageism Questionnaire for Nurses based on the standardized process of psychometric scale development, social exclusion theory, and practical experiences in nursing work. The questionnaire comprises two dimensions: “Ignore of elderly patients” and “Rejection of elderly patients.” It meets the validation standards for psychometric scales and demonstrates scientific rigor and operability in practical applications.

During the item development phase, this study employed Social Exclusion Theory as the theoretical foundation for constructing the Ageism Questionnaire for Nurses [34]. To ensure the scale reflects the practical characteristics of nursing work, in addition to gathering opinions from relevant experts, this study conducted structured interviews with nurses involved in geriatric care. This process resulted in the initial item pool, which included two dimensions (“Ignore of elderly patients” and “Rejection of elderly patients”) and 34 items. To ensure the scientific rigor, precision, and authenticity of the items, this study further invited 10 relevant experts from universities and hospitals in Jilin Province to conduct a Delphi evaluation. The high response rate and authority coefficient of the experts reflect the representativeness and professionalism of the Delphi evaluation in this study [45]. After two rounds of revisions, 8 items were excluded, leaving 26 items, all of which met relevant statistical criteria, further enhancing the scientific validity of the items in this study.

During the psychometric validation phase, this study conducted factor analysis and assessed the reliability and validity of the 26-item Ageism Questionnaire for Nurses. During exploratory factor analysis (EFA), three items exhibited factor loadings greater than 0.4 on both the “Neglect of elderly patients” and “Rejection of elderly patients” dimensions and were therefore removed. Confirmatory factor analysis (CFA) was then conducted on the remaining 23 items, with results indicating that all indices met the statistical requirements for CFA, demonstrating a good fit between the structural model and empirical data. In the reliability and validity testing [46], the total score of the Ageism Questionnaire in Nursing, the “Neglect of elderly patients” subscale, and the “Rejection of elderly patients” subscale were significantly positively correlated with ageism attitudes, while they were significantly negatively correlated with nurses’ work engagement. Internal consistency was assessed using Cronbach’s α coefficient, with the α coefficients for “Neglect of elderly patients” “Rejection of elderly patients,” and the total ageism score exceeding 0.90 in this study [47]. In split-half reliability testing, the split-half reliability coefficients for “Ignore of elderly patients,” “Rejection of elderly patients,” and the total scale exceeded 0.85. Test-retest reliability, used to evaluate the stability of the scale, showed coefficients for “Ignore of elderly patients,” “Rejection of elderly patients,” and the total ageism score exceeding 0.85, highlighting the stability of this questionnaire [48].

In the medical field, ageist attitudes toward elderly patients can easily lead to various issues that disrupt the orderly conduct of nursing work [49]. Numerous studies indicate that ageism toward elderly patients reflects individuals’ stereotypes about old adults, such as perceiving them as difficult to communicate with, unable to care for themselves, or having poor hygiene [50]. In the nursing domain, ageism is also evident in the growing care demands of elderly patients and the increasing pressure on nursing staff [51, 52],

Ageism toward elderly patients not only compromise the quality of nursing care but also undermine the overall effectiveness of healthcare delivery [49]. Numerous studies indicate that ageism toward elderly patients reflects individuals’ stereotypes about old adults, such as perceiving them as difficult to communicate with, unable to care for themselves, or having poor hygiene [50]. Ageism are exacerbated in nursing contexts, where staff face growing patient loads and increasingly complex care demands [51, 52].

Guided by Social Exclusion Theory, this study operationalized the Ageism Questionnaire for Nurses (AQN) across two dimensions: neglect and rejection of elderly patients. The key distinction lies in neglect representing passive inattention during care—a form of “unintentional exclusion” characterized by unconscious oversight rather than deliberate action (e.g.,In my nursing work, I accidentally overlook the needs that elderly patients express). This covert discrimination often remains undetected. Systemic pressures in aging societies may exacerbate such neglect in clinical practice [53]. In contrast, rejection manifests as active exclusion in clinical practice, reflecting more direct and intense socially discriminatory attitudes—a deliberate form of ageism that is typically overt and easily observable (e.g., “In my nursing work, I get irritated because of elderly patients’ chronic illnesses, which makes me show a rejecting attitude toward their care”). Distinguishing these two dimensions allows the AQN to capture a continuum of ageist behaviors that extends from subtle omissions in care delivery to explicit social distancing and avoidance, thereby providing a more comprehensive assessment of nurses’ discriminatory practices in clinical settings [39, 54, 55].

In the context of global aging, providing high-quality care for older adults presents a significant challenge. As central figures in care delivery, nurses’ performance is influenced by multifaceted factors spanning individual, cultural, and systemic dimensions. This study aims to explore one critical aspect of this complex phenomenon-ageism-with the goal of supporting nurses in better addressing the challenges associated with elder care. Unlike existing scales on ageism, which often lack contextual specificity to healthcare settings, this study focuses specifically on ageism exhibited by nursing staff during clinical practice. In summary, the Ageism Questionnaire in Nursing developed in this study builds upon previous theoretical research on ageism, further enriching its specific manifestations in nursing practice. In practical applications, this scale can assist nursing managers in more precisely evaluating nurses’ attitudes toward elderly patients, thereby facilitating targeted interventions. In the field of nursing education, it can also be used as a tool to provide specific training for students engaged in geriatric clinical nursing.

This study has several limitations that warrant further exploration in future research. First, the Ageism Questionnaire in Nursing developed in this study is a self-report scale, which may be subject to social desirability bias, potentially leading to less objective results. Future research could build on these findings to develop a questionnaire assessing elderly patients’ perceptions of ageism among nurses, as well as a scale for nursing managers to evaluate ageism among nursing staff. Second, the primary participants in this study were from Northeast China, and cultural and regional factors may have influenced the results. Future studies could expand upon these findings to further refine the scale and conduct reliability and validity testing across diverse cultural backgrounds, enhancing its applicability across cultures and regions.Third, the initial development of our scale items primarily relied on expert perspectives and literature analysis, lacking sufficient direct involvement of old adults. This limitation may affect the instrument’s applicability in real-world clinical settings. Future research should incorporate the views and experiences of old adults. when developing ageism assessment tools.Fourth, it should be noted that while we employed the Delphi method with statistically significant Kendall’s W coefficients in both rounds, the second round yielded a lower coefficient (*W* = 0.153, *p* < 0.05). This reduction may reflect experts’ heightened focus on nuanced item evaluation during the second assessment. To enhance objectivity and scientific rigor, subsequent studies should consider increasing the number of expert discussion and consultation rounds.

## Conclusion

In this study, the Ageism Questionnaire for Nurses (AQN) was developed, comprising 23 items across two dimensions: elderly neglect and elderly rejection. The results of exploratory factor analysis (EFA) and confirmatory factor analysis (CFA) demonstrated satisfactory structural validity of the scale. Additionally, the psychometric evaluation confirmed that the criterion validity, internal consistency reliability, split-half reliability, and test-retest reliability of the scale met established psychometric standards. The AQN serves as a valid and reliable instrument for assessing ageism among nursing staff in geriatric care settings.

## Supplementary Information

Below is the link to the electronic supplementary material.

Supplementary Material 1

## Data Availability

The data that support the findings of this study are available from the corresponding author.
