# Supplementary material for: Development and psychometric properties of the Ageism Questionnaire for Nurses (AQN)
Source: BMC Nurs. 2026 Aug 6;25:1057. doi: 10.1186/s12912-026-05198-3 (PMC13729350; doi:10.1186/s12912-026-05198-3)
Supplement: Supplementary file 1 [file 12912_2026_5198_MOESM1_ESM.docx]

### **Supplementary material 1**

### **Table 1.Ageism Questionnaire Interview Outline**

Gender: Age: Department: Education background:

| Number | Interview Outline |
| --- | --- |
| 1 | How do you understand ageism? |
| 2 | In your nursing work, have you or your colleagues exhibited ageist behaviors? |
| 3 | What specific manifestations of ageist behaviors have you observed in your work? |
| 4 | In your daily nursing practice, do you approach the care of elderly patients differently compared to patients from other age groups? If so, could you describe these differences in terms of communication, clinical decision-making, or resource allocation? |
| 5 | Can you recall any particularly challenging or adverse incidents while providing care to elderly patients? Please describe the circumstances and how they were resolved. |
| 6 | During such difficult encounters, aside from patient-related factors, what personal reactions or emotional responses did you experience? How did these influence your subsequent interactions with elderly patients? |

### **Supplementary material 2**

### **Table 2. Characteristics of the Delphi Expert Panel (N=12)**

| **ID** | **Professional Background** | **Academic Qualification** | **Years of Experience** | **Primary Work Setting** | **Area of Expertise** | **Response** |
| --- | --- | --- | --- | --- | --- | --- |
| E1 | Nursing Professor | Ph.D. in Nursing (China) | 8 | University School of Nursing | Geriatric nursing education; elderly care research | Accepted |
| E2 | Nursing Professor | Ph.D. in Nursing (China) | 7 | University School of Nursing | Chronic disease management in older adults | Accepted |
| E3 | Nursing Professor | Ph.D. in Nursing (China) | 6 | University School of Nursing | Aging and health promotion; nursing intervention | Accepted |
| E4 | Nursing Professor | Ph.D. in Nursing (China) | 6 | University School of Nursing | Dementia care; long-term care quality evaluation | Accepted |
| E5 | Nursing Administrator | Master's in Nursing(China) | 15 | Tertiary Hospital Nursing Department | Clinical nursing management; geriatric ward operations | Accepted |
| E6 | Nursing Administrator | Master's in Nursing(China) | 15 | Tertiary Hospital Nursing Department | Elderly patient safety; nursing quality improvement | Accepted |
| E7 | Nursing Administrator | Master's in Nursing(China) | 15 | Tertiary Hospital Nursing Department | Nursing human resources; geriatric care protocols | Accepted |
| E8 | Nursing Administrator | Master's in Nursing(China) | 12 | Tertiary Hospital Nursing Department | Inpatient care coordination; elderly services | Accepted |
| E9 | Psychology Professor | Ph.D. in Psychology (China) | 6 | University Department of Psychology | Mental health in later life; aging attitudes | Accepted |
| E10 | Psychology Professor | Ph.D. in Psychology (China) | 5 | University Department of Psychology | Social cognition in aging; age-related stereotypes | Accepted |
| E11 | Psychology Professor | Ph.D. in Psychology (China) | 4 | University Department of Psychology | Emotional well-being of older adults; caregiver psychology | Declined |
| E12 | Psychology Professor | Ph.D. in Psychology (China) | 4 | University Department of Psychology | Older adults with cognitive impairment; mental health services | Declined |
